# Tezosentan reduces the microvascular filtration coefficient in isolated lungs from rats subjected to cecum ligation and puncture

**DOI:** 10.1186/cc3882

**Published:** 2005-10-18

**Authors:** Vladimir Kuklin, Mikhail Sovershaev, Thomas Andreasen, Vegard Skogen, Kirsti Ytrehus, Lars Bjertnaes

**Affiliations:** 1 Research fellow, Department of Anaesthesiology, Faculty of Medicine, University of Tromsø, MH building, 9037 Tromsø, Norway; 2 Research fellow, Department of Physiology, Faculty of Medicine, University of Tromsø, MH building, 9037 Tromsø, Norway; 3 Departmental engineer, Department of Physiology, Faculty of Medicine, University of Tromsø, MH building, 9037 Tromsø, Norway; 4 Associate professor, Department of Internal Medicine, University Hospital of Tromsø, MH building, 9037 Tromsø, Norway; 5 Professor, Department of Physiology, Faculty of Medicine, University of Tromsø, MH building, 9037 Tromsø, Norway; 6 Professor, Chairman of the Department of Anaesthesiology, Faculty of Medicine, University of Tromsø, MH building, 9037 Tromsø, Norway

## Abstract

**Introduction:**

We recently demonstrated that the non-selective endothelin-1 (ET-1) receptor blocker tezosentan antagonizes ovine acute lung injury (ALI) following infusion of endotoxin or ET-1 by reducing the enhanced lung microvascular pressure, although we could not exclude the possibility of a simultaneous decline in microvascular permeability. In the present study, our aim was to find out if tezosentan reverses the rise in microvascular filtration coefficient (Kfc) in rat lungs that have been isolated and perfused 12 h after cecum ligation and puncture (CLP) or infusion of ET-1.

**Methods:**

Wistar rats (n = 42) were subjected to CLP. Postoperatively, rats were randomized to a CLP group (n = 7) and a CLP + tezosentan group (n = 7); the latter received tezosentan 30 mg/kg. A sham-operated group (n = 5) underwent laparotomy without CLP. Twelve hours postoperatively, the lungs were isolated and perfused with blood from similarly treated rats that also were used to assess plasma concentration of ET-1 and protein kinase Cα (PKCα) in lung tissue. Additionally, isolated blood perfused lungs from healthy rats were randomized to a control group (n = 8), an ET-1 group (n = 7) subjected to pulmonary arterial injection of ET-1 10 nM, and an ET-1 + tezosentan group (n = 7) that received tezosentan 30 mg/kg. All lung preparations received papaverine 0.1 μg/kg added to the perfusate for vasoplegia. Pulmonary hemodynamic variables, Kfc and lung compliance (C_L_) were assessed.

**Results:**

After CLP, the plasma concentration of ET-1 increased. Papaverine abolished the vasoconstrictor response to ET-1 and the pulmonary vascular pressures remained close to baseline throughout the experiments. Both CLP and injection of ET-1 caused significant changes in Kfc and C_L _that were prevented in tezosentan-treated rats. Compared to sham-operated animals, CLP increased the content of PKCα by 50% and 70% in the cytosolic and the membrane fractions of lung tissue homogenates, respectively. Tezosentan prevented the upregulation of PKCα in the membrane fraction.

**Conclusion:**

In rat lungs isolated and perfused after CLP, tezosentan precludes both the increase in Kfc and the upregulation of PKCα in the membrane fraction of lung tissue.

## Introduction

The potent vasoconstrictor peptide endothelin-1 (ET-1) is released in response to sepsis and endotoxemia [1,2]. Recent investigations have shown that in rats subjected to cecum ligation and puncture (CLP) the plasma concentration of ET-1 increases until a maximum has been reached 10 to 12 h after the surgical intervention [3,4].

When administered to the pulmonary circulation of healthy rats, ET-1 causes leukocyte adhesion, platelet aggregation and histological changes consistent with interstitial lung edema [5,6]. In isolated rat lungs in which the vasculature has been paralyzed with papaverine, injection of ET-1 into the pulmonary artery provokes pulmonary edema, but the mechanisms involved are not fully understood [7].

In the cell, activation of protein kinase C alpha (PKCα) is supposed to be an integral part of the signal transduction system of ET-1 [8-10]. Studies *in vitro *have revealed that activation of PKCα, which includes translocation from cell cytosol to the membrane, contributes to increased endothelial permeability [11,12]. Based on these observations, investigators have hypothesized that in the lungs activation of PKCα might cause changes that could result in acute lung injury (ALI) [13]; however, to our knowledge this hypothesis has not been tested in any study of lungs from septicemic animals.

We recently reported experiments in sheep in which the ET-1 receptor antagonist tezosentan attenuates endotoxin-induced ALI, as evaluated by a decline in extravascular lung water [14]. In that investigation, tezosentan reduced extravascular lung water by lessening the pulmonary microvascular pressure. Additionally, we noticed that tezosentan decreases the slope of the regression line between extravascular lung water and microvascular pressure, but its effect on microvascular permeability could not be determined [15]. We also found that tezosentan prevents the activation of PKCα in lung tissue [15]. Thus, we speculate whether tezosentan, in addition to its dampening effect on lung microvascular pressure, also counteracts the increase in microvascular permeability by preventing activation of PKCα in lung endothelial cells.

The aims of the present study were: first, to investigate if rats subjected to CLP respond with increased plasma levels of ET-1, alterations in PKCα in lung tissue and an enhanced lung fluid filtration coefficient (Kfc); second, to find out if administration of ET-1 to blood perfused lungs isolated from healthy rats induces the same kind of changes; and finally to find out if tezosentan attenuates the observed changes in PKCα and Kfc induced by CLP or administration of ET-1.

## Methods

The study was performed according to the Helsinki Convention for Use and Care of Animals and with the approval of the Norwegian Experimental Animal Board.

### Surgical procedures

Male Wistar rats (n = 154) weighing 250 to 350 g were used. For surgical intervention, rats were anesthetized with a combination of fentanyl and fluanisone (Hypnorm^®^, Janssen Pharmaceutica, Beerse, Belgium) and midazolam (Dormicum^®^, F Hoffman-La Roche AG, Basel, Switzerland) at a dose of 0.01 to 0.05 mg per 100 g and 1.0 to 1.75 mg per 100 g, respectively. Three experimental groups were used. In the CLP group (n = 7), rats underwent CLP as previously described [16,17]. Briefly, cecum was isolated via a midline laparotomy, ligated at a point corresponding to 35% of its average length, punctured twice with a 13-gauge needle, and compressed to extrude bowel contents into the peritoneum. The abdominal wound was closed in two layers and infiltrated with bupivacaine (Marcain^®^, AstraZeneca AS, Oslo, Norway) 1 ml (2.5 mg) for postoperative analgesia. Postoperatively, saline (3 ml per 100 g body weight) was injected subcutaneously. In the CLP + tezosentan group (n = 7), rats were additionally treated with tezosentan (Actelion Ltd, Allschwil, Switzerland) 30 mg/kg dissolved in saline (3 ml per 100 g body weight). The sham-operated group (n = 5) only underwent laparatomy. The laparotomy was closed as described above and saline was given as for the CLP groups. In each experiment, we used four similarly treated animals. After 12 h with free access to food and water, one rat underwent lung isolation and perfusion and two were used as blood donors. The fourth was used for determination of PKCα in lung tissue homogenates and sampling of blood for testing of bacterial growth and analysis of the plasma concentration of ET-1.

### Lung isolation

Lungs of all the three groups were prepared as previously described [7,18]. Briefly, rats were anesthetized, tracheotomized and ventilated at 70 inflations/minute employing tidal volumes (V_TD_) of 2 ml and positive end-expiratory pressure (PEEP) of 2.0 cmH_2_O. The chest was opened with a median sternotomy. Heparin (Nycoheparin^®^, Leo Pharma AS, Oslo, Norway) 250 IU dissolved in 1.0 ml saline was injected into the right ventricle. Then, the heart-lung preparation was removed, cannulated, and perfused at constant flow inside a thermostated chamber (38°C) using a roller pump (2115 Multiperpex LKB, Bromma, Sweden). Air was evacuated by perfusing briefly with Krebs-Ringer solution, which was subsequently replaced by 20 ml of autologuous whole blood obtained by heart puncture of two similarly treated rats. Heparin 100 IU was added to each 10 ml of blood. The perfusate was pumped from a reservoir via the pulmonary artery, and re-circulated via a cannula in the left atrium. The cannula was connected to a ladder-like tube allowing left atrial outflow pressure to be intermittently raised. Pulmonary arterial pressure (P_PA_) and left atrial pressure (P_LA_) were measured with pressure transducers (Transpac III; Abbott, North Chicago, IL, USA) via T-shaped side-ports in the pulmonary artery cannula and in the left atrial cannula, distal to the ladder, as described previously [18]. Perfusate flow was increased gradually until a pulmonary artery pressure of approximately 20.5 cmH_2_O was reached corresponding to a constant flow of 10 to 15 ml/minute, as determined at the end of the experiment.

Ventilation was with the same settings as above, and airway pressure (P_AW_) was monitored with a pressure transducer (Transpac III; Abbott). All the pressures were recorded on a Gould 6600 polygraph (Gould Instruments, Valley View, OH, USA). Gas containing 21% oxygen, 5% carbon dioxide save nitrogen was supplied from a Douglas bag.

### Measurements and calculations

Lungs were suspended in a weight transducer (FT 30C, Grass Instruments, Quincy, MA, USA) that was connected to the polygraph to allow continuous measurement of the lung weight. The Kfc was determined as described by previous investigators [19]. Briefly, after an isogravimetric state was obtained, lungs were subjected to an elevation of P_LA _of 7.88 cmH_2_O by clamping the lower step of the ladder for a period of 6 minutes every 30 minutes during the 120 minute experiment to provide conditions for fluid filtration. Pulmonary microvascular pressure (Pmv) was measured during elevation of P_LA _and at baseline using the double vascular occlusion method [20]. The resulting increase in Pmv (ΔPmv) was calculated as the difference between Pmv during elevation of P_LA _and at baseline. The weight gain curve displayed a biphasic pattern, with an initial steep part, which is due to a rise in intravascular blood volume during elevation of P_LA_, followed by a flatter part, which is caused by fluid filtration [21]. The rate of weight gain (in g/minute) during elevation of P_LA _was averaged over the last 4 minutes of the lung weight gain curve and used to calculate Kfc according to the formula Kfc = ΔW/4/ΔPmv. All Kfc values were normalized to 100 g predicted lung weight (P_LW_), which was based on body weight (B_W_) according to P_LW _= 0.0053 B_W _- 0.48 and expressed as ml/minute/cmH_2_O per 100 g [19,22]. Total vascular resistance (R_T_) was calculated as R_T _= (P_PA _- P_LA_)/Q (where Q is perfusate flow (ml/minute)) and lung compliance (C_L_) as C_L _= V_TD_/P_AW _– PEEP.

### Experimental protocols

To verify vascular paralysis, isolated blood-perfused lungs from healthy rats (n = 4) were subjected to injections of ET-1 10 nM (Sigma Chemical, St Louis, MO, USA) into the pulmonary arterial tubing before and after the injection of papaverine 0.1 μg/kg (Norges Apotekerforening AS, Oslo, Norway).

All the lung preparations isolated from CLP- and sham-operated rats received a pulmonary arterial injection of papaverine 0.1 μg/kg from the onset of perfusion. The CLP + tezosentan group additionally received tezosentan 30 mg/kg added to the perfusate. The other groups received a corresponding volume of the solvent.

To study the effect of tezosentan on ET-1-induced lung injury, isolated blood-perfused lungs from healthy rats received papaverine 0.1 μg/kg and were subsequently randomized to: a control group (n = 8); an ET-1 group (n = 7), which received an injection of ET-1 10 nM into the pulmonary artery; an ET-1 + tezosentan group (n = 7) subjected to injection of ET-1 as above, and with the addition of tezosentan 30 mg/kg after 5 minutes. The preparations underwent the same elevations of P_LA_, and measurements and calculations were the same as described above. After termination, lungs were stored in liquid nitrogen for later assessment of PKCα.

### Microbiology

Right ventricular blood (1 ml) was collected aseptically, inoculated in standard blood culture bottles (aerobic and anaerobic) and incubated in an automated system (BacT ALLERT 3D, Organon Technica, Durham, NC, USA). Identification of microbial growth was performed according to standard methods.

### Western blotting

PKCα was assessed as previously described [15]. Briefly, samples were homogenized in ice-cold extraction buffer (250 mmol/l sucrose, 1 mmol/l EDTA, 1 mmol/l EGTA, 20 mmol/l Tris-HCl pH 7.5, 10 mmol/l 2-mercaptoethanol, 20 mmol/l dithiothreitol and 1 tablet of Complete^® ^EDTA-free protease inhibitor cocktail (Roche Diagnostics GmbH, Mannheim, Germany) per 10 ml), centrifuged at 200 × g to remove debris followed by 100,000 × g for 60 minutes at 4°C. The supernatant was collected (cytosolic fraction), and the pellet resuspended by sonication in buffer supplemented with 1% TritonX-100 and centrifuged at 25,000 × g for 15 minutes at 4°C to obtain the soluble membrane fraction. For SDS-PAGE, 10% polyacrylamide gels were loaded with 10 mg of protein per lane. Membranes were probed with anti-PKC-α primary antibodies (Santa Cruz Biotechnology, CA, USA). A ChemiLucent detection kit (Chemicon, Temecula, CA, USA) was used in combination with a Kodak Image Station 1000 (Kodak, Rochester, NY, USA) for densitometry readings.

### Determination of ET-1

Plasma concentrations of ET-1 were determined with ELISA (R&D Systems Inc., Minneapolis, MN, USA) according to the manufacturer's instructions.

### Statistical analysis

Data are expressed as mean ± standard error of the mean (SEM). The data were assessed by two-factor ANOVA for repeated measurements using SPSS 11.0 for Windows (LEAD Technologies Inc, Chicago, IL, USA). If *F *value was statistically significant, Scheffe's test was used for *post hoc *inter-group analysis. Test of contrasts was used to evaluate differences within groups towards baseline (time 0 minute). One-way ANOVA was used to evaluate differences in PKCα between groups. P < 0.05 was considered statistically significant.

## Results

Polymicrobial Gram-positive and/or Gram-negative bacterial growth was found in six of seven blood cultures from the CLP group and five of seven rats in the CLP + tezosentan group. No growth was found in blood cultures from sham-operated rats.

### Vascular reactivity to ET-1

**Figure 1:**
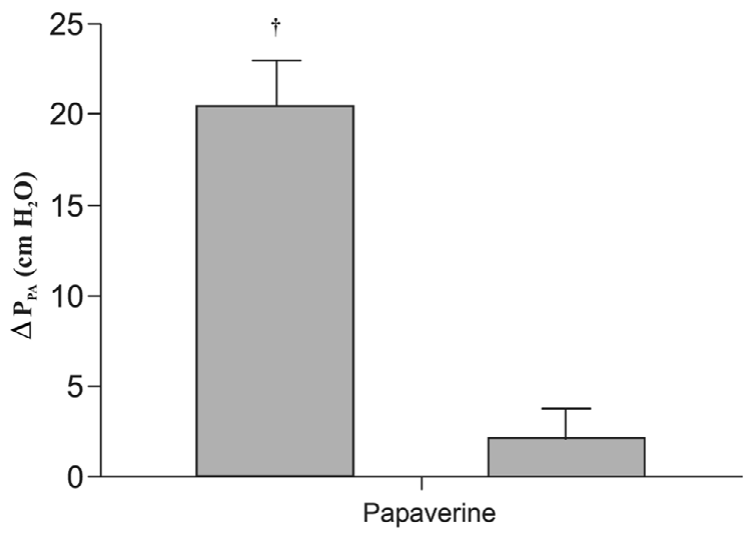
Pulmonary arterial pressure responses (ΔP_PA_) to endothelin-1 (ET-1) before and after papaverine administration in isolated lungs. Data are presented as mean ± SEM. ^†^p < 0.05 from baseline.

Injection of ET-1 into the pulmonary arterial tubing increased P_PA _by 115% from baseline (p < 0.05; Figure 1). Administration of papaverine restored P_PA _to a level close to baseline (time 0). Further injections of ET-1 did not cause any significant changes in P_PA_.

### CLP-induced pulmonary edema

**Figure 2:**
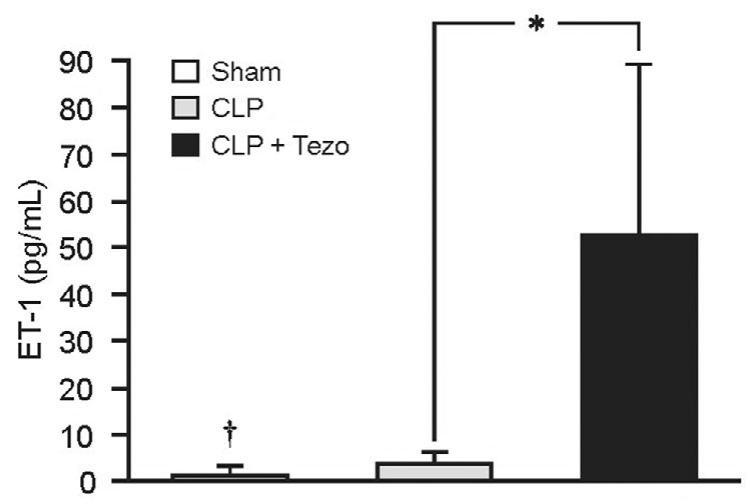
Plasma concentrations of endothelin-1 (ET-1) in rats determined 12 h after surgical interventions. Data are presented as mean ± SEM. Sham, sham-operated group (n = 5); CLP, cecum ligation and puncture group (n = 7); CLP+Tezo, cecum ligation and puncture + tezosentan group (n = 7). *p < 0.05 between CLP and CLP+Tezo groups; ^†^p < 0.05 between Sham and CLP groups.

CLP induced a fourfold increase in the plasma concentration of ET-1 compared to sham-operated rats (p < 0.05; Figure 2). However, in the CLP + tezosentan group, the plasma level of ET-1 was 10 to 15 times higher than with CLP alone (p < 0.05).

**Figure 3:**
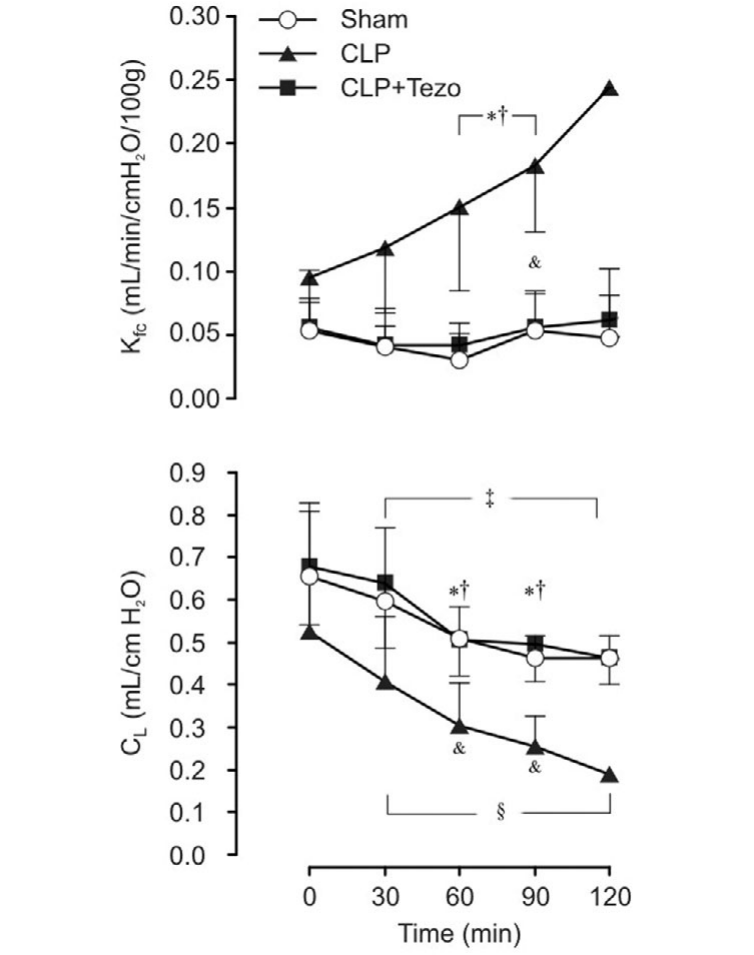
Microvascular filtration coefficient (Kfc) and compliance (C_L_) in lungs isolated 12 h after surgical interventions. Data are presented as mean ± SEM. Sham, sham-operated group (n = 5); CLP, cecum ligation and puncture group (n = 7); CLP+Tezo, cecum ligation and punction + tezosentan group (n = 7). *p < 0.05 between CLP and CLP+Tezo groups; ^† ^p < 0.05 between Sham and CLP groups; ^‡ ^p < 0.05 from t = 0 minutes in Sham group; ^&^p < 0.05 from t = 0 minutes in the CLP group; ^§^p < 0.05 from t = 0 minutes in the CLP+Tezo group.

At baseline, we found no differences in hemodynamic variables between sham-operated rats and the CLP groups (Table 1). Because of the papaverine-induced vascular paralysis, hemodynamics displayed no intra- or inter-group differences throughout the experiments. In sham-operated rats, Kfc displayed no difference between groups at baseline and remained unchanged throughout the experiment (Figure 3). At variance, a threefold increase was noticed in the CLP group. Concomitantly, C_L _decreased fourfold in parallel with increasing pulmonary edema beyond 30 minutes. All preparations deteriorated with visible fluid secretion into the airways after 90 minutes of perfusion (p < 0.05; Figure 3). In contrast, in the CLP + tezosentan group, Kfc remained unchanged from baseline throughout the experiment and C_L _displayed no significant difference from sham-operated animals.

### ET-1-induced pulmonary edema

**Figure 4:**
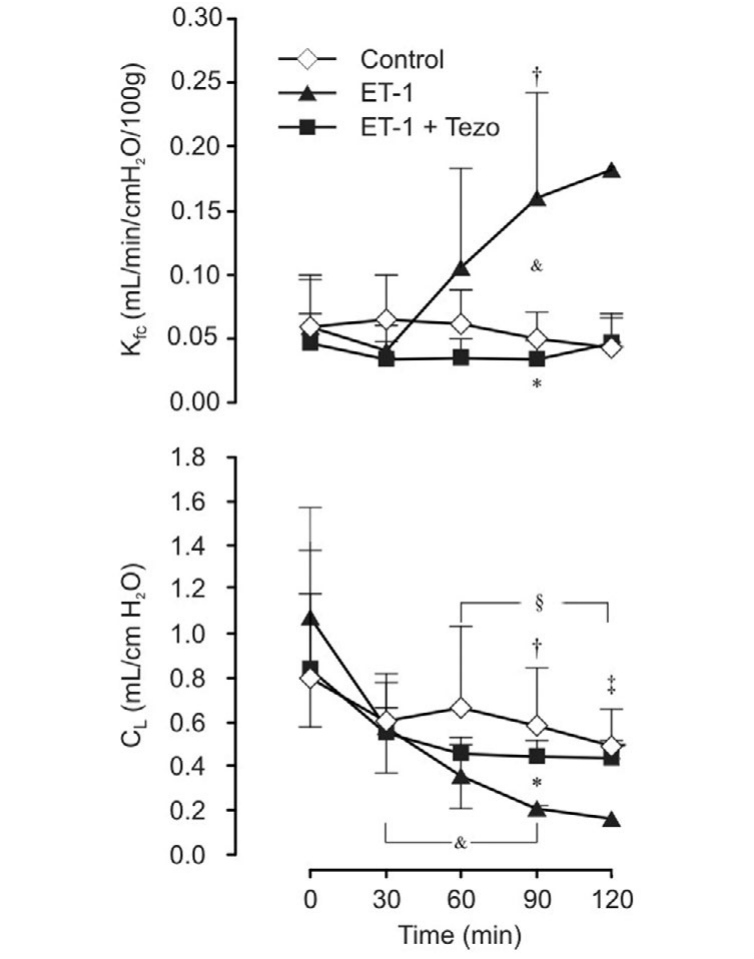
Microvascular filtration coefficient (Kfc) and compliance (C_L_) after endothelin-1 (ET-1) administration in isolated lungs from healthy rats. Data are presented as mean ± SEM. Control, control group (n = 8); ET-1, endothelin-1 group (n = 7); ET-1+Tezo, endothelin-1+tezosentan group (n = 7). *p < 0.05 between ET-1 and ET-1+Tezo groups; ^†^p < 0.05 between control and ET-1 groups; ^‡^p < 0.05 from t = 0 minutes in the control group; ^&^p < 0.05 from t = 0 minutes in the ET-1 group; ^§^p < 0.05 from t = 0 minutes in the ET-1+Tezo group.

Injection of ET-1 into the pulmonary arterial tubing caused a significant rise in Kfc at 90 minutes, which was completely prevented by tezosentan (p < 0.05; Figure 4). All preparations exposed to ET-1 alone, except for one, were completely destroyed after 90 minutes due to alveolar flooding. Administration of tezosentan maintained Kfc at baseline level throughout the experiments. Correspondingly, C_L _fell in all three groups. In the ET-1 group, C_L _decreased fivefold compared to the intra-group baseline (p < 0.05; Figure 4). The decrease was significantly dampened in the ET-1 + tezosentan group and did not differ from control lungs. Hemodynamic variables revealed no significant differences between the groups (Table 2).

### PKCα in lung tissue after CLP or ET-1

**Figure 5:**
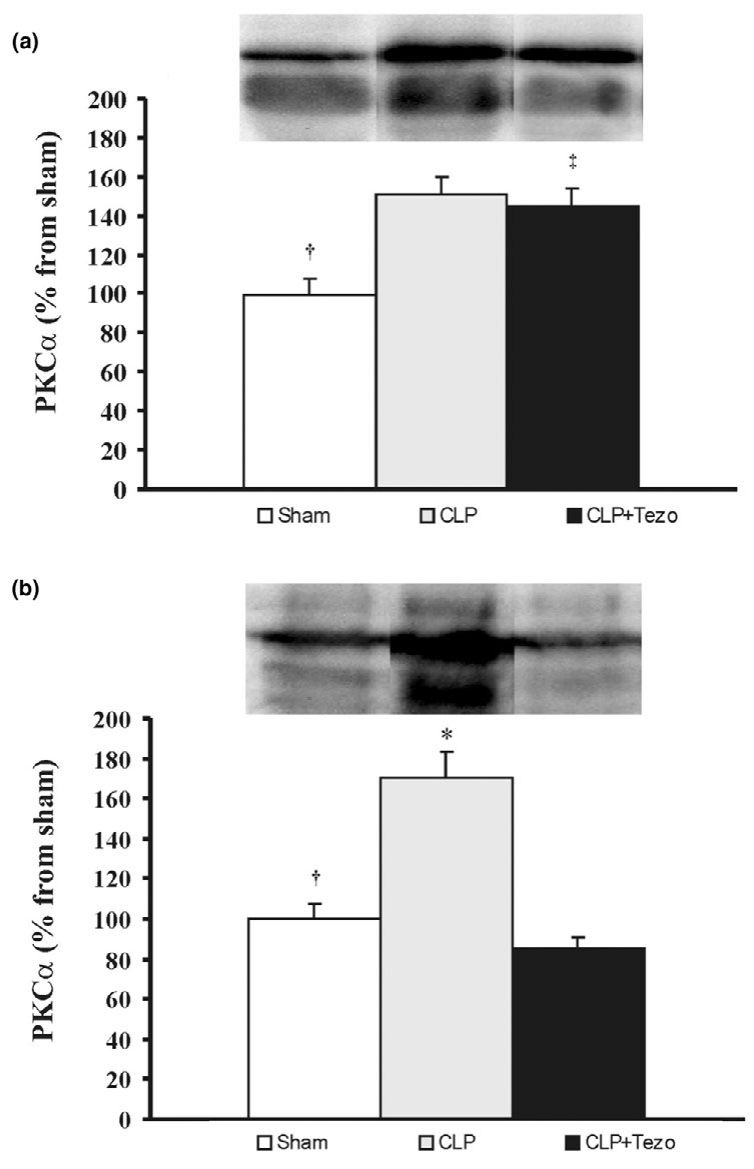
Protein kinase Cα in lungs after surgical interventions. **(a) **Cytosolic and **(b) **membrane fractions. Data are presented as mean ± SEM. Sham, sham-operated group (n = 5); CLP, cecum ligation and puncture group (n = 6); CLP+Tezo, cecum ligation and puncture + tezosentan group (n = 6). ^†^p < 0.05 between Sham and CLP groups; ^‡^p < 0.05 between Sham and CLP+Tezo groups; *p > 0.05 between CLP and CLP+Tezo groups.

In the CLP group, the immunoreactivity of PKCα reached a mean of 50% to 70% above sham in both tissue fractions (p < 0.05; Figure 5). Tezosentan completely prevented the rise in the cell membrane fraction of PKCα (Figure 5b).

**Figure 6:**
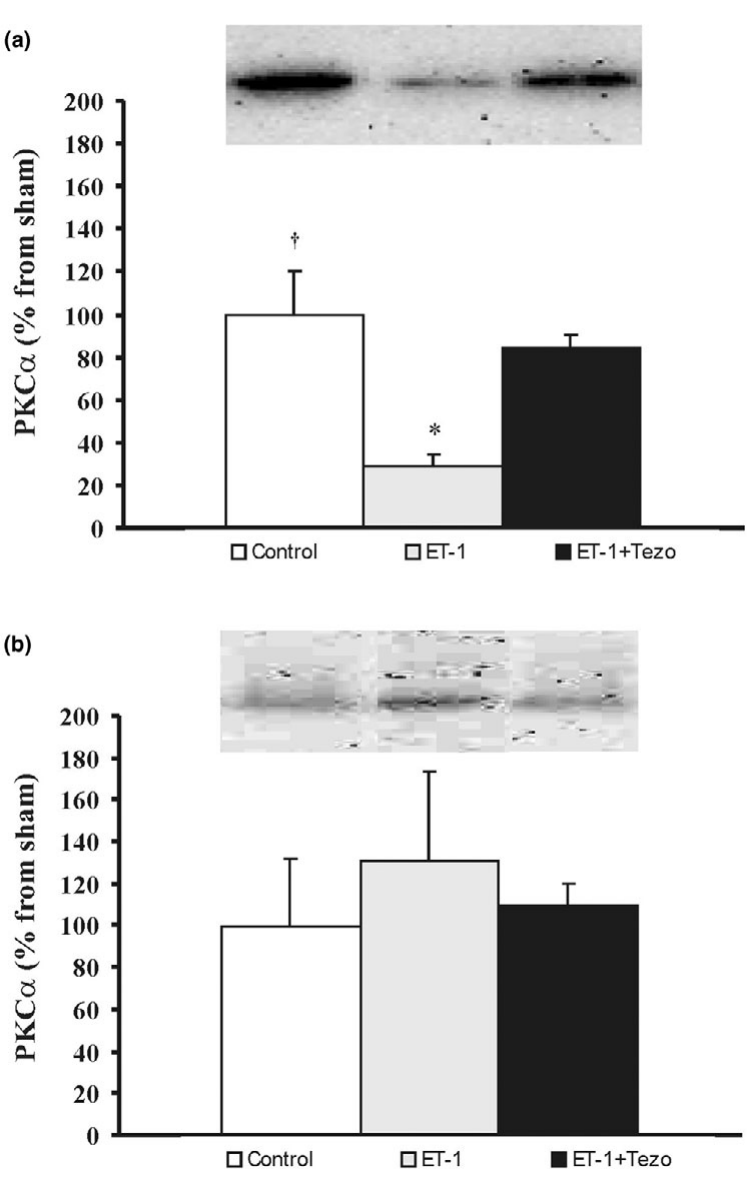
Protein kinase Cα in lungs after endothelin-1 (ET-1) administration. **(a) **Cytosolic and **(b) **membrane fractions. Data are presented as mean ± SEM. Control, control group (n = 4); ET-1, endothelin-1 group (n = 4); ET-1+Tezo, endothelin-1+tezosentan group (n = 4). ^†^p < 0.05 between control and ET-1 groups; *p > 0.05 between ET-1 and ET-1+Tezo groups.

In lungs isolated from healthy rats, acute administration of ET-1 decreased the cytosolic fraction of PKCα by 60% (p < 0.05; Figure 6a) and correspondingly tended to increase (not significant) the cell membrane fraction compared to controls (Figure 6b). Moreover, tezosentan prevented the reduction of the cytosolic fraction of PKCα (p < 0.05; Figure 6a).

## Discussion

The present study demonstrates that in rats CLP induces a significant rise in the plasma concentration of ET-1 in parallel with an increase in the PKCα content of lung tissue. Lungs isolated and perfused with blood 12 h after CLP displayed visible edema fluid in the trachea before 120 minutes had elapsed. Correspondingly, in lungs isolated from healthy rats, pulmonary arterial injection of ET-1 produced massive edema within 60 minutes of the start of blood perfusion. Tezosentan precluded the development of pulmonary edema induced by both CLP and ET-1. As judged by western blotting, tezosentan also prevented the increase in PKCα in lung tissue after CLP. Thus, we speculate that ET-1-binding to the endothelin receptor could be responsible either for promoting PKCα gene expression and protein synthesis, or for inhibiting PKCα degradation.

When assessing changes in microvascular permeability in response to ET-1 or other vasoconstrictors, papaverine is used to deprive the lungs of their vasoconstrictor ability, which implies that the Pmv can be kept constant [7,18,23]. The control group confirmed that papaverine had no effect on lung microvascular permeability *per se *as previously demonstrated [7,23]. Consistent with these findings, papaverine prevented ET-1-induced changes in pulmonary arterial pressure, but did not preclude the evolvement of pulmonary edema. In lungs from sham-operated or healthy rats, in which no intervention had taken place except for the injection of papaverine, Kfc remained unchanged for the whole 120 minute perfusion time.

Other investigators have noticed significant increments in Kfc and protein concentration in lung lavage fluid 18 h after CLP in isolated rat lungs [24]. There is, however, no general agreement about what factors determine the morbidity and mortality after CLP. Some investigators argue that mortality depends on the size of the punctured holes in the cecum [16]. Others claim that increased length of the cecum distal to the ligature raises the plasma concentrations of tumor necrosis factor-α and interleukin-6, both factors that might contribute to the high mortality during the first 16 to 24 h [17]. By combining the two techniques, we expected that changes in Kfc would develop at a higher pace. Consistently, we found that rats subjected to our modification of CLP appeared ill and less vigorous in comparison with sham-operated animals. Moreover, the modified CLP, but not sham-operation, displayed growth of Gram-negative and Gram-positive microorganisms in rat blood.

Several factors might contribute to the development of pulmonary edema after CLP in rats [24,25]. Both experimental and clinical studies have shown that transient increases in the plasma concentrations of ET-1 might be associated with development of pulmonary edema [2,14,26-29]. In patients diagnosed with ALI, derangement of pulmonary function was exacerbated by elevated plasma concentrations of ET-1, whereas clinical improvement was associated with a significant fall in concentrations of ET-1, indicating that ET-1 could act as a marker of ALI [26-28]. In other species, however, ET-1 participates in several other pathophysiological mechanisms besides being a marker of vascular injury [29,30]. In rats, continuous infusion of ET-1 resulted in escape of ^125^I-labelled albumin to liver, heart and lungs while hematocrit increased [31]. At doses of 5 to 10 nM, ET-1 caused pulmonary edema in isolated rat lungs perfused with salt solution while no change was observed when a blood perfusate was used [32-34]. After pre-treatment with ibuprofen, however, ET-1 increased the pulmonary microvascular permeability during blood perfusion [34]. Employing a fluorescent technique, the investigators demonstrated that ET-1 reduced the filtration area by two thirds, whereas after ibuprofen the lungs were fully perfused [34]. Consistent with a previous investigation [7], we found that ET-1 at a dose of 10 nM increased microvascular permeability in blood-perfused lungs in which the vasculature had been paralyzed. It seems to us that paralyzed vasculature is a pre-requisite for equal distribution of ET-1 and its effects on permeability. Depressed vascular reactivity to angiotensin II and KCl has been reported recently in lungs isolated from rats after CLP [35]. In that investigation, activation of inducible nitric oxide synthase (iNOS) with enhanced production of NO in lung tissue was assumed to cause vascular hyporeactivity [35]. We did not check for expression of iNOS in the present study, but as the vasculature was paralyzed by papaverine after baseline measurements, we doubt that NO-induced vasodilatation has contributed to a further enlargement of the filtration area.

In the present study, we noticed that CLP increased the plasma concentration of ET-1, and lung edema developed shortly after perfusion was started. We also observed that non-selective ET-1 receptor blockade completely prevented edema. These findings are consistent with a recent observation of prevention of ET-1 or lipopolysaccharide-induced microvascular leakage in the airways after ET-1 receptor subtype A (ET_A_) receptor blockade in rats [36]. In contrast to our study, however, these investigators studied animals *in vivo *and did not control pulmonary microvascular hydrostatic pressure.

We noticed that in septicemic rats, the plasma concentration of ET-1 was significantly lower than the minimum concentration required for increasing pulmonary microvascular permeability in healthy rats [37]. Actually, we doubt that the plasma level reflects the concentration of ET-1 in lung tissue. The latter suggestion is partly supported by the observation of enhanced plasma concentrations of ET-1 after administration of tezosentan (Figure 2). Previous investigators have suggested that big ET-1 is converted to active ET-1 in the lungs [38]. Accordingly, others have noticed that intravenously injected big ET-1 increases the extravasation of Evans blue in lung parenchyma of healthy rats whereas blockade of ET-1 converting enzyme with phosphoramidon prevents the leakage [37]. Researchers studying cecum perforation in rats observed that the concentration of ET-1 and big ET-1 in peritoneal fluid increased to 400 pg/ml 12 h after surgery [39]. In contrast, the simultaneously measured total ET-1 concentration in plasma amounted to 81 pg/ml only. This slow increase in the plasma level in spite of a high local concentration could, in part, be due to the fact that ET-1 is secreted from the abluminal surface of the endothelial cells [40]. Additionally, endothelins are rapidly cleared by the lungs [41]. Frelin *et al*. [42] suggest that the endothelins bind stoichiometrically to receptors, which means that most ligand molecules are bound to receptors and, therefore, cannot be determined in plasma, albeit that even low concentrations of circulating endothelins may be biologically active [42]. As suggested by recent investigators, competition at the receptor between ET-1 and its antagonists could result in release of ET-1 from the receptor, thereby contributing to an overall increase in the plasma concentration consistent with the present findings [43].

Little is known about the mechanism by which ET-1 influences microvascular permeability, and what additional mediators might be involved. We recently reported that in sheep an apparent association exists between endotoxin-induced ALI and activation of PKCα in the lungs [16]. Consistently, tezosentan both prevented ALI and attenuated the activation of PKCα. In the present rat model of sepsis-induced lung injury, PKCα expression was markedly upregulated, but tezosentan prevented a part of this upregulation. This also corresponded with the prevention of edema in isolated lungs. In ALI induced by ET-1, we noticed a reduced trend towards translocation and activation of PKCα after tezosentan. The present study demonstrates a difference in PKC involvement between ET-1 and CLP-induced ALI. As judged from our results with tezosentan, ET-1 seems to be involved both in the activation and production of PKC in the lungs. However, further studies are warranted to fully elucidate the effects of non-selective ET-1 receptor blockade on activation of PKCα and its influence on the integrity of lung microvasculature.

## Conclusion

In rats subjected to CLP, increased plasma levels of ET-1 are associated with changes in lung microvascular permeability. Apparently, these changes are linked to activation of PKCα in lung tissue homogenates. Administration of ET-1 to lungs isolated from healthy rats mimics the CLP-induced changes in permeability, but not in the activation of PKCα. Finally, tezosentan ameliorates CLP and ET-1 induced increases in microvascular permeability and prevents activation of PKCα in lung tissue of septicemic rats.

## Key messages

• 	In rats, CLP increases the plasma concentration of ET-1 and activates PKCα in lung tissue.

• 	Lungs with a paralyzed vasculature that were isolated and perfused with whole blood 12 h after CLP developed fulminant edema before 120 minutes had elapsed.

• 	Correspondingly, in lungs isolated from healthy rats, in which the vasculature had been paralyzed, injection of ET-1 into the pulmonary artery induced pulmonary edema within 60 minutes.

• 	The non-selective ET-1 receptor blocker tezosentan prevents both CLP- and ET-1-induced pulmonary edema in isolated blood perfused rat lungs.

• 	Tezosentan also precludes CLP-induced activation of PKCα in lung tissue.

## Abbreviations

ALI = acute lung injury; CLP = cecum ligation and puncture; ET-1 = endothelin-1; Kfc = microvascular filtration coefficient; P_AW _= airway pressure; PEEP = positive end-expiratory pressure; PKCα = protein kinase C alpha; P_LA _= left atrial pressure; Pmv = pulmonary microvascular pressure; P_PA _= pulmonary arterial pressure; V_TD _= tidal volume.

## Competing interests

This study was supported by Helse Nord (Norway), project number 4001.721.132 and departmental funds of the Departments of Anaesthesiology and Physiology, University of Tromsø, Norway. The authors declare that they have no competing interests.

## Authors' contributions

VK participated in the design of the study, analyzed the data, and drafted the manuscript. MS, TA, VS and KY contributed with biochemical analyses, microbiological investigation and participated in the design of the study. LB administered the study, participated in the design of the study and suggested improvements to the manuscript. All authors read and approved the final manuscript.

## Figures and Tables

**Table 1:** Hemodynamic variables in rat lungs isolated 12 h after surgical interventions

Hemodynamic variable	Time (minutes)				
	0	30	60	90	120

P_PA_, cmH_2_O					
Sham	26.0 ± 3.1	24.6 ± 3.1	24.6 ± 2.7	26.0 ± 3.0	26.0 ± 3.0
CLP	21.9 ± 1.5	23.2 ± 1.3	23.2 ± 1.6	24.6 ± 2.3	
CLP+Tezo	24.6 ± 0.9	23.2 ± 0.9	23.2 ± 1.1	21.9 ± 1.2	24.6 ± 1.7
P_LA_, cmH_2_O					
Sham	14.6 ± 0.6	14.7 ± 0.6	14.9 ± 0.9	15.6 ± 0.9	14.9 ± 0.9
CLP	13.8 ± 0.4	13.8 ± 0.4	13.8 ± 0.4	13.4 ± 0.8	
CLP+Tezo	14.9 ± 0.4	14.9 ± 0.4	14.7 ± 0.4	15.4 ± 0.4	15.2 ± 0.4
R_T_, cmH_2_O/ml/min					
Sham	0.89 ± 0.1	0.80 ± 0.1	0.83 ± 0.1	0.84 ± 0.1	0.82 ± 0.1
CLP	0.57 ± 0.1	0.65 ± 0.1	0.67 ± 0.1	0.67 ± 0.1	
CLP+Tezo	0.75 ± 0.0	0.67 ± 0.0	0.67 ± 0.1	0.67 ± 0.1	0.71 ± 0.1
ΔPmv, cmH_2_O					
Sham	7.2 ± 0.2	7.2 ± 0.3	7.5 ± 0.3	7.2 ± 0.3	7.5 ± 0.3
CLP	6.7 ± 0.5	6.5 ± 0.4	6.4 ± 0.4	6.5 ± 0.6	
CLP+Tezo	7.6 ± 0.4	7.6 ± 0.4	7.5 ± 0.4	8.0 ± 0.4	7.6 ± 0.5

Data are presented as mean ± SEM. Sham, sham-operated group (n = 5); CLP, cecum ligation and puncture group (n = 7); CLP+Tezo, cecum ligation and puncture + tezosentan group (n = 7). P_LA_, left atrial pressure; P_PA_, pulmonary artery pressure; ΔPmv, difference between pulmonary microvascular pressure determined prior to and during a standardized elevation of P_LA_; R_T_, total vascular resistance.

**Table 2:** Hemodynamic variables in blood perfused lungs isolated from healthy rats

Hemodynamic variable	Time (minutes)				
	0	30	60	90	120

P_PA_, cmH_2_O					
Control	22.0 ± 1.6	21.7 ± 1.7	22.4 ± 2.1	24.1 ± 1.9	23.4 ± 2.0
ET-1	19.7 ± 1.0	18.7 ± 1.3	18.9 ± 1.7	17.5 ± 2.0	
ET-1+Tezo	21.7 ± 0.4	21.0 ± 1.6	22.6 ± 1.2	19.8 ± 1.3	22.6 ± 1.6
P_LA_, cmH_2_O					
Control	14.3 ± 0.8	14.6 ± 0.9	14.6 ± 0.9	14.9 ± 0.8	14.9 ± 0.8
ET-1	13.1 ± 0.8	13.1 ± 0.9	13.1 ± 0.9	14.7 ± 1.2	
ET-1+Tezo	13.1 ± 0.8	13.1 ± 0.9	12.3 ± 0.8	10.9 ± 0.8	12.4 ± 0.8
R_T_, cmH_2_O/ml/min					
Control	0.58 ± 0.1	0.54 ± 0.1	0.60 ± 0.1	0.69 ± 0.1	0.72 ± 0.1
ET-1	0.49 ± 0.1	0.42 ± 0.1	0.43 ± 0.1	0.20 ± 0.1	
ET-1+Tezo	0.80 ± 0.1	0.75 ± 0.1	0.78 ± 0.0	0.68 ± 0.1	0.78 ± 0.1
ΔPmv, cmH_2_O					
Control	6.5 ± 0.4	6.5 ± 0.4	6.5 ± 0.4	6.5 ± 0.4	6.9 ± 0.4
ET-1	6.9 ± 0.4	6.9 ± 0.4	6.7 ± 0.4	6.9 ± 0.5	
ET-1+Tezo	7.2 ± 0.4	7.2 ± 0.4	7.2 ± 0.4	7.2 ± 0.4	7.3 ± 0.4

Data are presented as mean ± SEM. Control, control group (n = 8); ET-1, endothelin-1 group (n = 7); ET-1+Tezo, endothelin-1+tezosentan group (n = 7). P_LA_, left atrium pressure; P_PA_, pulmonary artery pressure; ΔPmv, difference between pulmonary microvascular pressure determined prior to and during a standardized elevation of P_LA _(7.9 cmH_2_O); R_T_, total vascular resistance.
